# When Plaquing Is Not Possible: Computational Methods for Detecting Induced Phages

**DOI:** 10.3390/v15020420

**Published:** 2023-02-02

**Authors:** Taylor Miller-Ensminger, Genevieve Johnson, Swarnali Banerjee, Catherine Putonti

**Affiliations:** 1 Bioinformatics Program, Loyola University Chicago, Chicago, IL 60660, USA; 2 Department of Mathematics and Statistics, Loyola University Chicago, Chicago, IL 60660, USA; 3 Department of Biology, Loyola University Chicago, Chicago, IL 60660, USA; 4 Department of Microbiology and Immunology, Stitch School of Medicine, Loyola University Chicago, Maywood, IL 60153, USA

**Keywords:** prophage, induction, temperate phages, metagenomics, genomics

## Abstract

High-throughput sequencing of microbial communities has uncovered a large, diverse population of phages. Frequently, phages found are integrated into their bacterial host genome. Distinguishing between phages in their integrated (lysogenic) and unintegrated (lytic) stage can provide insight into how phages shape bacterial communities. Here we present the Prophage Induction Estimator (PIE) to identify induced phages in genomic and metagenomic sequences. PIE takes raw sequencing reads and phage sequence predictions, performs read quality control, read assembly, and calculation of phage and non-phage sequence abundance and completeness. The distribution of abundances for non-phage sequences is used to predict induced phages with statistical confidence. In silico tests were conducted to benchmark this tool finding that PIE can detect induction events as well as phages with a relatively small burst size (10×). We then examined isolate genome sequencing data as well as a mock community and urinary metagenome data sets and found instances of induced phages in all three data sets. The flexibility of this software enables users to easily include phage predictions from their preferred tool of choice or phage sequences of interest. Thus, genomic and metagenomic sequencing now not only provides a means for discovering and identifying phage sequences but also the detection of induced prophages.

## 1. Introduction

Phages are the most abundant biological entities on Earth [1]. Recent investigations into the human microbiota have found that, here too, phages outnumber both cellular organisms and eukaryotic viruses (see review [2]). High-throughput sequencing of the human virome has uncovered phages that have yet to be characterized, a.k.a. the ‘dark matter’ [3,4]. Phages colonize all anatomical sites of the human body (see review [5]). The human virome has been cataloged most extensively in the gut [6], and to a lesser extent in other anatomical sites, e.g., oral cavity [7], urinary tract [8], skin [9].

Typically, phages persist in these communities through one of two life cycles: lysogeny and lysis. In the lysogenic life cycle, the phages integrate into the bacterial host genome (or persist as an extrachromosomal plasmid) and replicate with the bacterial cell. Integrated phage genomes (prophages) can be a substantial proportion of bacterial genomic content (see review [10]). Most bacteria in the human microbiota are lysogens, harboring one or more prophages [11,12,13,14,15,16]. In contrast, during the lytic life cycle, phages replicate within the bacterial host cell, producing mature phages that lyse (kill) the host cell, thus dispersing mature phages into the surrounding environment. Temperate phages are capable of persisting through both of these life cycles, “induced” from the lysogenic life cycle into the lytic life cycle often via an external cue or spontaneously (see review [17]). This switch in life cycles has captured the interest of microbiologists for decades; several different methods have been developed and explored in the laboratory to induce temperate phages [18,19,20,21,22,23,24,25,26,27]. Furthermore, models describing the dynamics of temperate phages have been proposed and contested (see review [28]). These temperate phages can have a profound effect on a microbial community’s diversity, and within the human microbiome this can be related to symptoms/disease, e.g., Crohn’s disease [29].

Identifying and characterizing temperate phages at the bench necessitates a susceptible host for propagation. Phages are often noted as having narrow host ranges, capable of lysing a single species or even specific strains [30]. As such, identifying a host susceptible to the reproduction of a temperate phage through the lytic life cycle is far from trivial. In lieu of plaque assays, phage and prophage sequences have been identified from metagenomic and genomic data using bioinformatic tools, e.g., VirSorter2 [31], PHASTER [32], VIBRANT [33], and Cenote-Taker 2 [34]. These tools, however, are not capable of ascertaining the lifecycle of the phage.

The concept of phage-to-host (PtoH) aims at discerning between phages in the lytic and lysogenic life cycles [35]. Briefly, in high-throughput sequencing data sets, the lysogenic phage (prophage) copy number should be equivalent to the bacterial genome copy number (ratio ≈ 1) as it is integrated into the bacterial genome and should be replicated at the same rate. In contrast, lytic phages should have a ratio >1. When a phage reproduces via the lytic cycle, a few to hundreds of phage progeny are produced [36]. Thus, we would expect to see a higher copy number of induced lytic phages than that of the bacteria and an integrated phage, causing a shift in the PtoH ratio. Furthermore, phages can degrade their host’s DNA [37], further shifting the PtoH ratio. Recently, this concept has been explored more broadly to predict phages that are in the lytic cycle from genomic and/or metagenomic data sets [38,39]. The ability to predict inducible prophages using PtoH ratios has been proven experimentally on several species of bacteria, including *Salmonella enterica*, as well as on fecal samples from mice [40]. Additionally, induced prophages have been identified in metagenomes of human and murine gut samples as well as peatland soil metagenomes [35,39].

Here we present the Prophage Induction Estimator (PIE). Similar to these recently released tools, PIE uses the prophage genome copy number and bacterial genome copy number to detect induced prophages. Confidence in these predictions comes from bootstrapping the copy number (coverage) of individual bacterial contigs and estimating a distribution of such coverage values. In silico tests were performed to benchmark this new tool. Additionally, we examined single genome raw data to identify an induced phage. We also conducted sequencing for a mock community of seven bacterial strains and examined it as well as a urinary metagenome in an effort to identify induction events in communities. From sequence data alone, induced prophages can be detected, circumventing the need of a naïve host to ascertain a prophage’s ability to enter the lytic cycle.

## 2. Materials and Methods

### 2.1. Bacterial Strains, Genome Sequencing, and Genome Assembly

Seven bacterial isolates were selected for this study; all seven were previously isolated using the expanded quantitative urine culture method (EQUC) [41] from a single urine sample (Loyola University Chicago, Maywood, IL, USA, IRB # 206469) [41], and obtained from Dr. Alan J. Wolfe (Loyola University Chicago, Maywood, IL, USA). These isolates include *Escherichia coli* UMB1284, *Actinomyces neuii* UMB1295, *Staphylococcus hominus* UMB1296-1T, *Lactobacillus jensenii* UMB1303, *Enterococcus faecalis* UMB1309, *Proteus mirabilis* UMB1310, and *Corynebacterium amycolatum* UMB1310-1E.

Bacteria from freezer stocks were streaked onto CNA Blood agar plates and incubated at 35 °C and 5% CO_2_ for 18–36 h. A single colony from each plate was grown in 1 mL of the bacterium’s respective medium (Table 1) for 18–36 h at 35 °C and 5% CO_2_. DNA extraction and sequencing of some of these strains has been previously described: *E. coli* UMB1284 [42], *A. neuii* UMB1295 [43], *L. jensenii* UMB1303 [44], *E. faecalis* UMB1309 [45], and *P. mirabilis* UMB1310 [46]. For *E. coli* UMB1284, DNA was extracted using the Qiagen DNeasy UltraClean Microbial Kit following the manufacturer’s protocol, and libraries were constructed using the Nextera XT DNA Library preparation kit and sequenced on the Illumina MiSeq platform (MiSeq Reagent Kit v2 (500-cycles)) at Loyola University Chicago’s Genomics Facility (Maywood, IL, USA) [42]. For the remaining strains, including the two strains sequenced as part of this study (*S. hominus* UMB1296-1T and *C. amycolatum* UMB1310-1E), bacterial cultures were extracted using a modified version of the Qiagen Blood and Tissue Kit Protocol. (For details regarding modifications, see [43,45,46].) DNA concentrations were quantified using a Qubit fluorometer following the manufacturer’s protocol. Samples were then shipped to MIGS (Pittsburgh, PA, USA). There, sequencing libraries were prepared using the Illumina Nextera Kit and samples were sequenced using the Illumina NextSeq 550 platform. Raw reads from the Illumina sequencing were trimmed via Sickle (https://github.com/najoshi/sickle) and assembled using SPAdes v.3.14.1 [47]. Genome coverage was computed using BBmap (https://jgi.doe.gov/data-and-tools/bbtools/). Raw sequencing reads and assembled genomes are available in NCBI’s SRA and Assembly databases, respectively (Table 1). This includes the depositing of the sequencing reads and assemblies for *S. hominus* UMB1296-1T and *C. amycolatum* UMB1310-1E, sequenced as part of this study.

### 2.2. Prophage Sequence Prediction

Prophage sequences were predicted using PHASTER [32] via the webserver. PHASTER predicts phage sequences as Intact, Questionable, and Incomplete. Sequences from all three PHASTER phage prediction categories were included in our analyses.

### 2.3. Culturing Bacteria for Spontaneous Induction and Sequencing

A community of all bacterial strains was created as follows. Each bacterial strain was grown in 13 mL of their respective media (Table 1) for ~18 h, except for *Corynebacterium amycolatum* UMB1310-1E, which was grown for ~36 h. All cultures were grown at 35 °C and 5% CO_2_. A total of 1 mL of the overnight culture was added to 1 mL of fresh media and 2 mL of nuclease free water and grown overnight, ~18 h, at 35 °C, and in 5% CO_2_. All seven cultures were pooled into a centrifuge tube and filtered using vacuum filtration with a sterile 0.22 um cellulose acetate membrane screw-top filter (Corning Life Sciences, Corning, NY USA). A Macrosep tube (Pall, Port Washington, NY, USA; Omega Membrane 100K) was used to concentrate the filtrate. The Macrosep tube was prepared using 70% ethanol followed by 5% Tween 20 (Sigma-Aldrich, St. Louis, MO, USA) washes. Samples were processed using the Macrosep tube as follows: (1) Add up to 10 mL of sample to the Macrosep tube, (2) spin at 4000× *g* until ~300 uL of sample flowed through the filter and discard flow through, (3) repeat steps 1 and 2 until all of the sample is loaded into the tube and only 300 uL remains, and (4) gently scrape filter with a pipet tip and pipet concentrate to a clean microcentrifuge tube.

Concentrated filtrate was next DNased following the OPTIZYME DNase I Fisher Bioreagent’s protocol. DNA was extracted from the concentrated filtrate using the Zymo Research *Quick*-DNA Viral DNA Kit following the manufacturer’s protocol. DNA was amplified for sequencing using the Sigma GenomePlex Single Cell Whole Genome Amplification kit following the manufacturer’s protocol and then cleaned for sequencing using the Omega Bio-tek E.Z.N.A Cycle Pure Kit, again following the manufacturer’s protocol. Samples were sequenced using the Illumina NextSeq 550 platform at MIGS as previously described. Sequencing reads are available in NCBI’s SRA database, accession No. SRR18907418.

### 2.4. Mock Community Construction

For each of the seven genomes, the raw reads were subsampled to generate four data sets representative of four different genome coverages: 0.01×, 0.1×, 1×, and 10×. The size of the assembled genome and the read length was used to calculate the number of read pairs sampled. The PHASTER phage sequence predictions were used to generate read data at 10× and 100× coverages. ART (v.MountRainier-2016-06-05) was used to generate these Illumina read data sets (150 bp paired-end reads) (full parameter list: -ss HS25 -p -l 150 -f 10 [or 100] -m 1000 -s 200) [48]. Three simulated phage read data sets for each coverage were made. The first phage data set (“one_prophage”) includes just a single phage sequence predicted from *E. coli* UMB1284. The second phage data set (“some_prophage”) includes 10 phage sequences, randomly selected from the predicted phage sequences for the seven genomes. This data set includes the same phage included in the first set as well as another phage sequence from UMB1284, one predicted phage sequence from UMB1296-T, two predicted phage sequences from UMB1295, two predicted phage sequences from UMB1309, two predicted phage sequences from UMB1310, and one predicted phage sequence from UMB1310-1E. In the last phage data set (“all_prophage”), all 34 PHASTER predicted phage sequences for the seven bacterial assemblies were included. Then, to mimic possible PtoH ratios that could be found in high-throughput sequencing data, bacterial and phage reads were combined at varying ratios to produce sets of paired-end reads. For each of the three phage data sets, “one_prophage”, “some_prophage”, and “all_prophages”, five sets of paired-end reads were fabricated from the bacterial reads and the phage reads to represent a PtoH ratio of 10,000:1, 1000:1, 100:1, 10:1, and 1:1.

### 2.5. Identification of Positive Control Induced Phage

A strain of Pseudomonas aeruginosa, UMB2738, is known to contain a prophage that spontaneously induces in laboratory settings. Previously, the bacterial culture of UMB2738 and the isolated induced phage, Dobby, were both sequenced for analysis and characterization of the phage [49,50]. The genome of Dobby (GenBank Accession No. NC_048109.1) was used as the phage reference file necessary as input for PIE. The raw reads of UMB2738 (SRA Accession No. SRR9992785) also were input to PIE for analysis and prediction of induced phages.

### 2.6. Identification of Induced Phage in a Metagenome Data Set

The raw sequencing reads of a urinary metagenome sample was retrieved from GenBank (SRA Accession No. SRR19149281). This data represents DNA sequencing of a urine sample (sample ID 6162). In other words, both bacterial and viral constituents of the urinary microbiota are expected to be present. Details regarding the sample collection, DNA extraction, and sequencing was previously reported [51]. Briefly, a urine sample was obtained via transurethral catheterization of a female without lower urinary tract symptoms as part of a prior IRB-approved study (Loyola University Chicago, Maywood, IL, USA, IRB # 207102). A total of 10% by volume of AssayAssure^®^ was added to 2 mL of the collected urine and stored at −80 °C. DNA was extracted from the thawed urine sample using the Norgen Urine DNA according to the manufacturer’s protocol with one exception: a starting volume of 500 uL was used and the binding solution was adjusted accordingly. The DNA sequenced at MIGS (Pittsburgh, PA, USA) using the Illumina DNA Prep kit and IDT 10 bp UDI indices on an Illumina NextSeq 2000 (paired-end reads, 2 × 151 bp). This raw data set includes human reads; the urine was not processed prior to DNA extraction to remove human cells (if present). We thus removed human reads from further analysis using Bowtie 2 v.2.4.5 and the application’s publicly available human genome index (GRCh38) [52]. Only reads that did not map to the human genome were considered further. This included 1,403,038 read pairs, 56.90% of the raw data. These filtered sequencing reads were assembled using metaSPAdes v.3.15.4 [53] with default parameters. Assembled contigs were then evaluated by VirSorter2 to identify putative viral sequences [31], the results of which were queried against the NCBI nr/nt nucleotide database via megaBLAST (using default parameters) to identify the most similar sequence record. The filtered reads and predicted viral sequences were next analyzed using PIE. Viral sequences exceeding the 99% threshold in PIE were queried against the NCBI nr/nt nucleotide database via BLAST in an effort to identify their putative taxonomy and annotated via the Bacteriophage “annotation recipe” on BV-BRC v.3.28.9 [54].

### 2.7. Prophage Induction Estimator (PIE)

In addition to the functionality developed in Python (v3.9) and R, PIE integrates existing tools, including BBTools v.38.94 (https://jgi.doe.gov/data-and-tools/bbtools/), SPAdes v.3.15.3 (using SPAdes’ metaspades.py script) [47], and NCBI BLAST+ (https://ftp.ncbi.nlm.nih.gov/blast/executables/blast+/LATEST/). A Docker container was created for PIE. Source code and the Docker container can be obtained from https://github.com/putonti/PIE. Test data are also available through the Github Repository. PIE is also available through Docker Hub at: https://hub.docker.com/repository/docker/genevievej16/pie. The GitHub Repository provides information on installation, either via cloning the project from GitHub or via Docker Hub, as well as use and interpretation of results. Furthermore, test data, including the data used in our in silico study, are available through the GitHub Repository.

## 3. Results

### 3.1. Computational Method for Detecting Induced Prophages

Our method, Prophage Induction Estimator (PIE), distinguishes induced prophages from integrated prophages through differential read coverage. This approach assumes that an induced phage has a greater copy number (and thus read coverage) than its integrated form. In a culture of a single bacterial strain containing an inducible prophage, phage DNA is expected to be more abundant than its bacterial host’s DNA if: (1) induction leads to full (or near full) lysis of its bacterial host and/or (2) the culture contains bacterial hosts susceptible to the induced phage to replicate efficiently through the lytic life cycle. If neither of these two conditions occurs, distinguishing between phages in their induced and integrated form cannot easily be performed with confidence. PIE was designed to facilitate induced phage detection even when bacterial DNA is abundant. Furthermore, it can be applied to sequencing data generated from a single genome as well as simple or complex microbial communities.

**Figure 1:**
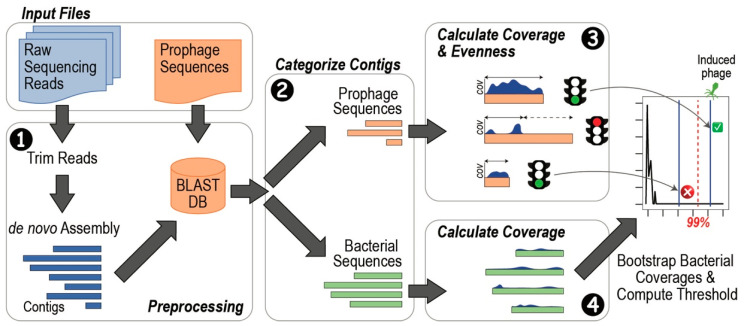
Schematic of PIE software. Required input files include raw sequencing reads (paired-end or single-end) and prophage sequence predictions (FASTA format file). Raw reads are trimmed and assembled (❶) and categorized via BLAST as prophage or bacterial in origin (❷). After categorization, sequence coverage is computed for both the prophage sequences (❸) and bacterial sequences (❹). For the prophage sequences, an additional metric is computed: evenness. Prophage sequences that do not have an evenness ≥90% are not considered further (red stop light). Prophage sequences meeting the evenness threshold (green stop light) are compared with the threshold for the distribution of bacterial contig coverage values (shown as 99% in the figure). Prophage sequences with a coverage exceeding the threshold are called as induced phages (“√” in green box); prophage sequences that do not meet this threshold are not called as induced phages (“X” in red circle).

Figure 1 summarizes the process for discriminating between induced prophages and integrated prophages.

Step 1: Preprocessing: Raw sequencing reads (either paired-end or single-end reads) and prophage sequences are supplied by the user. The raw reads are first trimmed using BBTools’ bbduk script and then assembled using metaSPAdes. Assembled contigs are trimmed, removing reads shorter than a given threshold. Prophage sequences can be known phage sequences or sequences from prophage prediction tools. The prophage sequences supplied by the user are used to make a BLAST database.

Step 2: Categorizing Contigs: Assembled contigs from Step 1 are compared with the BLAST database created in Step 1 and identified as either bacterial sequences (green) if there is no similarity to the database or contigs matching the prophage sequences supplied by the user (peach) if there is high similarity to the database. In the event that the contig contained a prophage sequence, the contig was split into two, excluding the prophage sequence. Categorization is repeated on this new set of contigs until no contigs contain both bacterial and prophage sequences.

Step 3: Calculating contig coverage and the evenness of coverage for prophage sequences: The raw reads are mapped back to each prophage contig (peach) using BBTools’ bbmap script. It is important to note that the entire read set is mapped to each prophage contig independently. The average coverage is computed for each contig. For the prophage contigs, an evenness of coverage, or the percentage of the prophage sequence for which reads were mapped, is also computed. A threshold for the evenness of coverage is applied; by default, this threshold is equivalent to 90% of the prophage sequence length. If prophage sequences meet this threshold they are considered further (green traffic light); if not, they are not considered further (red traffic light). This threshold removes contigs for which a small region, e.g., a single gene was detected.

Step 4: Calculating contig coverage for bacterial sequences: The raw reads are mapped back to the bacterial contigs (green) using BBTools’ bbmap script. The average coverage is computed for each contig.

Step 5: Identify prophage sequences with relative abundances greater than bacterial “background”: The coverage values for the bacterial contigs are used to create a bootstrap sample of size 10,000. We use this bootstrap sample to create a distribution and find the 99th percentile of distribution of bacterial contig coverage values. We thus expect most of the data, e.g., bacterial contigs and integrated prophages, to be below this threshold. Prophage contigs with an average coverage greater than or equal to this threshold, i.e., sequenced at a depth greater than or equal to sequencing of non-prophage regions of the bacterial genome, are predicted to be induced. The default value for this threshold is set to 0.99; the user can change this threshold by passing in a different parameter value when running the application. Note that a lower value of the threshold is less conservative and therefore lowers the confidence of prediction. If there are not five or more bacterial contigs, the bootstrap sample is not produced; all prophage sequences that passed the criteria of Step 4 are predicted to be induced. This threshold is equivalent to the minimum required sample size for density estimation of unidimensional data [55].

### 3.2. Assessing PIE’s Ability to Detect Induced Prophages on In Silico Data

**Figure 2:**
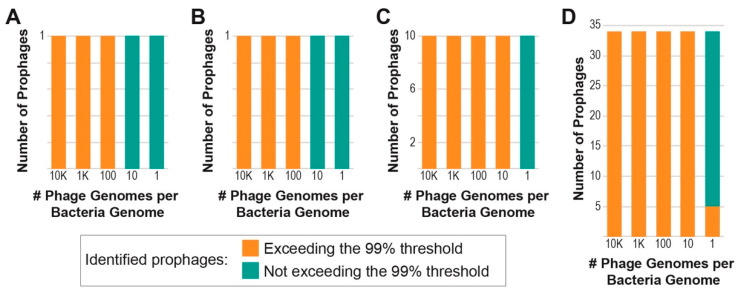
Induced prophages identified for a single bacterium (**A**) and mock communities (**B**–**D**). In the first test (**A**), a single prophage sequence with 100× genome coverage was added to bacterial reads producing a PtoH ratio of 10,000:1, 1000:1, 100:1, 10:1, and 1:1. (**B**) The same prophage sequence used in our prior test, again represented by reads at a 100× genome coverage, were added to reads from seven bacterial strains; each strain independently was sampled to produce the 5 different PtoH ratios tested. (**C**) A total of 10 prophage sequences and the seven bacterial genomes were examined at five different PtoH ratios. (**D**) 34 prophage sequences and the seven bacterial genomes were examined at five different PtoH ratios. For all charts, if the prophage(s) were identified and met/exceeded the 99% threshold, they are shown in orange. If they did not meet/exceed the threshold, they are represented by green.

We selected seven bacterial strains (Table 1) isolated from a single urine sample and thus members of a single community, for our in silico tests. Five of these strains were sequenced as part of our prior work [42,43,44,45,46], and two strains were sequenced as part of this study. Prophage sequences were predicted for the seven genomes using PHASTER. For each bacterium, we created four read sets representative of different genome coverages (see Methods): 0.01×, 0.1×, 1×, and 10×. Likewise, for our prophage sequences we created two synthetic read sets at 10× and 100×. For our first test, we evaluated PIE’s ability to detect an induced prophage from a single bacterium, *E. coli* UMB1284. This was simulated through the addition of prophage reads at 100× coverage to the 0.01×, 0.1×, 1×, and 10× bacterial read sets as well as the addition of prophage reads at 10× coverage to the 10× bacterial read set. These five read sets represent a PtoH of 10,000:1, 1000:1, 100:1, 10:1, and 1:1, respectively. As shown in Figure 2A, the one prophage was detected and met the 99% threshold when the bacterial genome coverage was significantly less than the prophage coverage. When the PtoH ratio was 10:1 and 1:1, the prophage sequence was identified but did not exceed the 99% threshold. Thus, PIE is efficient at detecting prophages with high confidence when the prophage coverage is greater than 10× the bacterial coverage.

Next, we considered communities of bacteria, including the read sets for the seven bacterial strains isolated from the same clinical sample. First, we assessed the ability to detect a single prophage, the same prophage used in our single bacterium test. As Figure 2B shows, the prophage was identified for all five data sets evaluated, but only exceeded the 99% threshold when the prophage coverage was greater than 10× the bacterial coverage. Next, we considered the bacterial community in the presence of multiple prophages. Ten different prophage sequences were added to the bacterial community read sets. All 10 of these prophages were detected, and with the exception of the PtoH of 1:1, all 10 of them exceeded the 99% threshold (Figure 2C). In our final test, all 34 prophages predicted for the seven bacterial strains were added to the bacterial community read sets (Sequences available). All 34 were identified and exceeded the 99% threshold for PtoH 10,000:1, 1000:1, 100:1, and 10:1. For PtoH 1:1, five of the 34 prophages exceeded the 99% threshold, while the remaining 29 did not (Figure 2D). When the threshold was reduced to 95%, 24 additional prophages (29 total) of the prophages exceeded this threshold. Sequences for both the 10 prophages and the 34 prophages can be found in the GitHub Repository’s “testFiles” directory.

### 3.3. Proof-of-Concept: Detecting Induced Phages

To assess the ability of PIE to detect induced phages in a single bacterial culture, we tested it on a previously studied strain of *P. aeruginosa*, UMB2738 [49]. *P. aeruginosa* UMB2738 is known to harbor a temperate phage, Dobby, that has been previously spontaneously induced, sequenced, and fully characterized [50]. We believed that because Dobby can spontaneously induce, it would be possible that the phage was also induced when the bacteria, UMB2738, was sequenced. The publicly available genome of Dobby was used as the phage reference file and the raw reads of UMB2738 were input to PIE (see Methods). Dobby was located in the UMB2738 reads by the tool and exceeded the 99% threshold. The average coverage for the Dobby sequence was 113.63×. This exceeded the bacterial contig average coverage, which was 43.56× (minimum 28.25×, maximum 90.23×).

**Figure 3:**
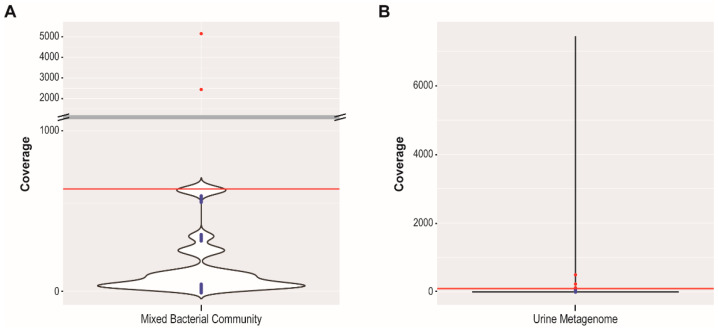
Density distribution of the coverage of the bacterial contigs from the (**A**) mixed community and (**B**) urine metagenome. The bacterial contig coverage values are shown by the density plot, where the width of the plot corresponds to the number of contigs with that coverage. The 99% threshold is shown by the red line. Predicted prophage sequences exceeding this threshold are shown as red circles, while predicted prophage sequences that do not meet this threshold are shown in blue circles. Density plots, such as those shown here, are automatically produced by PIE.

We next evaluated PIE’s ability to identify induced phages in mixed bacterial cultures; the seven bacterial strains used in our in silico analysis were grown in isolation. The cultures were pooled, filtered, DNA-extracted, amplified, and sequenced (see Methods). The resulting sequencing reads and the 34 PHASTER prophage sequence predictions for these seven bacterial strains were then processed using PIE. Sixty bacterial contigs (the largest 91 Kbp) and eight phage contigs were identified. Only two of the phages exceeded the 99% threshold, and in fact had coverage values exceeding all bacterial contig coverages, thus suggesting that they were induced in culture. These two phages include one from *E. coli* UMB1284 and one from *E. faecalis* UMB1309 (Figure 3A). PHASTER predicted the coliphage with a genome size of 24,504 bp as “Incomplete” and the *E. faecalis* phage with a genome size of 49,683 bp as “Intact” (File S1). The coliphage induced from UMB1284 exhibited the greatest sequence similarity to the metagenome-assembled genome (MAG) Myoviridae sp. Isolate ctYIP2 (GenBank Accession No. BK044152.1), with 67% query coverage and 93.97% sequence identity. The phage induced from *E. faecalis* UMB1309 most closely resembled the MAG Siphoviridae sp. Isolate ct4io3 (GenBank Accession No. BK02996.1; query coverage = 51% and sequence identity = 96.56%). Both of these MAGs were produced from human metagenomes [56]. Neither of the induced phage sequences exhibited sequence similarity with >1% query coverage to a GenBank record from an isolated phage.

In a final proof-of-concept, we evaluated PIE’s ability to identify putative induced phages from a real metagenome sample. As described in detail within the Methods, this metagenome was derived from a urine sample; as such, both bacterial and viral species present in the sample are expected to be represented in the DNA sequencing reads. A total of 88 putative viral sequences were predicted by VirSorter2 from this metagenome (average length 3273 bp) (File S2). Four of the VirSorter2 predicted sequences passed the 99% threshold for PIE (File S3). Figure 3B shows the density distribution of this analysis. Most of the bacterial contigs and VirSorter2 predicted sequences in this sample had low coverage values <100×. However, a few bacterial contigs had high coverage values, hence the narrow “spike” in the density distribution. We next queried the four sequences exceeding the 99% threshold against the NCBI nr/nt database to predict their taxonomy (Table 2).

## 4. Discussion

While lysogeny is prevalent within the human microbiome, the frequency of induction is unknown, and only a handful of mechanisms for induction within these communities have been identified [57,58]. Metagenomic sequencing led to the discovery of novel phages that have yet to be isolated in the laboratory [59,60,61,62,63,64]. Beyond identifying phages, the raw sequencing data can be used to shed light on induction events. Assuming induced phages outnumber their integrated copies, the PtoH ratio provides a means of distinguishing between phages in the lytic and lysogenic life cycle. As the in silico tests show, PIE identifies induced phages with high confidence (statistically significant) when there are >10× more phage genomes than bacterial genomes (Figure 2A,B). These two tests included just a single phage. When more phages were considered, high confidence predictions were made at the 10:1 (phage-to-host) ratio (Figure 2C) and some were even predicted at the 1:1 (phage-to-host) ratio (Figure 2D).

It is important to note that the in silico tests performed here used real sequencing reads for the bacterial isolates in this mock data set, instead of generating synthetic reads from their assembled genomes. As it is well-established that genome sequencing technologies do not produce uniform distributions of reads [65], sampling sequences from real read sets generates test data that are inherently biased. Our approach for identifying induced phages with confidence takes these biases into consideration. Non-phage sequence coverage values are sampled to generate a distribution of coverage values that reflect the variations in coverage depth along bacterial genomes. This contrasts the approaches of hafeZ [38] and PropagAtE [39], which also use PtoH ratios to identify induced phages. hafeZ uses the median coverage score while PropagAte uses the average coverage score to find regions with higher-than-expected coverage.

While both hafeZ and PropagAtE require raw reads and assembled genomes (in the case of both tools) or metagenome assemblies (in the case of PropagAtE) for analysis, PIE only requires raw reads as it performs read trimming and assembly as part of its pipeline; an assembly, however, can be provided to PIE if the user prefers. hafeZ also necessitates access to the Prokaryotic Virus Orthologous Groups (pVOGs) database [66]. hafeZ identifies regions with higher-than-expected coverage (as described above), performs gene calling, and compares gene calls to the pVOGs database; if a region contains genes homologous to pVOGs, it is predicted to be an induced prophage. As part of its pipeline, PropagAtE either performs phage sequence prediction using the tool VIBRANT [33], which uses the VOG database as well as KoFam and Pfam (along with other software dependencies), or users supply genome assembly coordinates for phage sequences. In contrast, PIE does not include database searches, rather users supply phage sequences. In doing so, the user can use any tool(s) for prophage prediction, e.g., VirSorter2 [31], PHASTER [32], VIBRANT [33], and Cenote-Taker 2 [34]. In the proof-of-concept test with the synthetic data sets and the mixed bacterial cultures, PHASTER was used to predict phages while VirSorter2 was used for the urinary metagenome data set, thus showing the versatility of phage prediction tools that can be used. Because users supply the phage sequences, users can provide a file with sequences to specifically mine for: (1) phages of interest that may or may not be present in their sample or (2) phage sequences that are known to be missed by most prophage prediction tools, e.g., inoviruses [60]. An advantage of PIE is its availability in Docker Hub; thus, users do not need to install software included in the pipeline nor have extensive knowledge of working within UNIX-based systems.

Our test on the reads generated for a single bacterium as well as a mixed community, which harbor multiple putative prophages, provides insight into PIE’s ability to detect prophage induction single genomes and small communities. In the investigation of *P. aeruginosa* UMB1738, we were able to detect the induced phage Dobby, which was previously characterized after spontaneous induction and plaquing on a naïve host [50]. When considering the mixed community, the same members as our in silico tests, the 34 prophage sequences predicted often showed little resemblance to isolated, sequenced phages. This mixed community included bacterial taxa for which very few phages have been characterized (if any). Many of these taxa have very specific culture conditions prohibiting us to culture them together and thus requiring them to be grown in isolation and then pooled together for processing. Furthermore, we were uncertain if any of these phages were inducible; it is for these reasons that we removed most bacterial cells and amplified DNA prior to sequencing. The two induced phages that exceeded the 99% threshold (Figure 3A), exhibited no significant sequence similarity to characterized phages. This is most significant given that one of these phages is a coliphage, probably the best characterized group of phages to date.

In our last proof-of-concept test, we applied PIE to a urinary metagenome data set. There was no pretreatment of the sample to select for viral constituents (e.g., via filtration, CsCl density gradient fractionation, amplification). Therefore, bacterial DNA is expected to be abundant within the sample. Four sequences, predicted by VirSorter2, were identified as exceeding the 99% threshold (Figure 3B). While one of these putative viral sequences (NODE_24) was representative of a phage, one was most similar to the eukaryotic virus JC polyomavirus (NODE_65), and one was most similar to a plasmid sequence (NODE_1047). It is important to note that all sequences were predicted by VirSorter2 to be viral. VirSorter2 was designed to identify eukaryotic viruses; thus, this proof-of-concept suggests that PIE can be used to identify high copy number eukaryotic viruses in a mixed community as well as phages. Nonetheless, PIE was able to identify a phage with an average coverage >215×. The GenBank record that is nearly identical to the phage sequence identified here, TPA: Siphoviridae sp. ctX581 (Table 2), was assembled from publicly available human metagenome read data [56], the same study that produced the sequences resembling the phages identified in our mixed bacterial cultures (Figure 3A). The last sequence that exceeded the 99% threshold, NODE_15, does not BLAST to a phage or viral sequence (Table 2). Because the annotation of this sequence predicted only 1 out of the 81 predicted coding regions as a phage gene, we do not believe that this is a phage. The results of this proof-of-concept highlight that using PtoH ratios can also be applied to identifying high-copy number plasmids or eukaryotic viruses, depending upon the input sequences provided by the user.

It is important to note that unlike the mixed bacterial community (Figure 3A), some of the bacterial contigs from the urinary metagenome had coverage values greater than the “viral” sequences meeting the 99% threshold. The statistical approach employed here enables PIE to identify putative induced or high copy number lytic phages, even in the presence of significant bacterial DNA, as shown in Figure 3B with a real data set. This confirms the sensitivity observed through our in silico tests.

While PIE enables users to specify a threshold other than the default of 99%, manual curation can be used to investigate phages at lower thresholds. While several other phages in the mixed community are sequenced at a coverage greater than most bacterial contigs observed, they do not exceed the 99% threshold imposed (Figure 3A). They may be representative of prophages spontaneously inducing at low levels within the culture or sequences that are inherently biased to be over-sequenced by short-read technologies. The user can further investigate these “borderline” phages by referring to the PIE output files. In particular, one of the output files lists both phage and bacterial contig coverage values, which also may lend itself to discovery of novel phages or viruses in a metagenome. Furthermore, investigation of this file may provide insight into Intact phages that were predicted as Incomplete (in the case of PHASTER) or low confidence (in the case of VirSorter and VirSorter2) because they were not assembled on a single contig; similar coverage values may indicate a single phage sequence. Thus, the PIE output has further utility in refining predicted prophage sequences. The 88 VirSorter2 predicted sequences from the urinary metagenome were queried against the nr/nt database, revealing several phage sequences that did not meet the 99% threshold (File S4). However, their low coverage suggests that they represent a prophage sequence.

Discerning between the life cycle in which a phage persists in a community is critical in furthering our understanding of their role in the community. Phage predation can drive bacterial evolution [67] and population structure (see review [68]). Temperate phages can also contribute to microbial evolution via horizontal gene transfer [69], including conferring fitness advantages to their host (see review [70]). Using tools such as PIE, metagenomic studies can extend their analysis beyond simply what phages are present and what these phages encode to now predict the life cycle of these phages. This additional information may provide insight into the role that a particular phage plays in microbial community dynamics.

## 5. Conclusions

Overall, the PIE software provides a lightweight, rapid means of identifying induced prophages or even high copy number lytic phages in a single isolate or community. The sensitivity observed through our in silico tests suggests that PIE can detect widespread induction events as well as phages with a relatively small burst size (10×). The flexibility of this software enables users to easily include phage predictions from their preferred tool of choice. Furthermore, users can include predictions from multiple tools or phage sequences of interest. The method employed for detecting induced prophages takes into consideration the inherent biases in modern sequencing, providing high confidence predictions. Thus, metagenomic sequencing now not only provides a means for discovering and identifying phage sequences but also the detection of induced prophages.

## Figures and Tables

**Table 1:** Strains included in this study.

Strain	SRA Accession No.	GenBank Assembly Accession No.	Culture Media
*E. coli* UMB1284	SRR7534266	GCA_003892355.1	LB
*A. neuii* UMB1295	SRR11441016	GCA_012030015.1	Actinomyces Broth
*S. hominus* UMB1296-1T	SRR14752304	GCA_018919365.1	TSB
*L. jensenii* UMB1303	SRR9695709	GCA_007786145.1	MRS + 5% Tween80
*E. faecalis* UMB1309	SRR11441014	GCA_012030535.1	BHI
*P. mirabilis* UMB1310	SRR11441013	GCA_012030515.1	LB
*C. amycolatum* UMB1310-1E	SRR14752303	GCA_018919345.1	LB

**Table 2:** Urinary microbiome VirSorter2 predictions exceeding the 99% threshold.

Contig ID	Length	Coverage	BLAST Best Hit			
			Description	Query Cov	%ID	Accession No.
NODE_65	9541	536.29	JC polyomavirus	100	97.33	LT615220.1
NODE_24	37847	215.32	TPA: Siphoviridae sp. ctX581	100	99.36	BK014883.1
NODE_1047	1563	115.85	*L. gasseri* plasmid pHL20_1	92	99.72	CP072179.1
NODE_15	61268	109.23	*Lactobacillus iners* KY ^1^	86	99.06	CP048798.1

^1^ Gene features included in this alignment includes only one predicted phage protein product.

## Data Availability

Raw sequencing reads and assembled genomes are publicly available through NCBI. Table 1 lists the accession numbers.

## Supplementary Materials

The following supporting information can be downloaded at: https://www.mdpi.com/article/10.3390/v15020420/s1, File S1: Detected induced phage sequences from mock community; File S2: Multi-FASTA format file with the 88 sequences predicted by VirSorter2 for urinary metagenome sample 6162; File S3: Multi-FASTA format file with the four sequences exceeding PIE’s 99% threshold; File S4: Taxonomy of the best BLAST hit for the sequences of the 88 VirSorter predictions.
